# MicroRNA-497 Reduction and Increase of Its Family Member MicroRNA-424 Lead to Dysregulation of Multiple Inflammation Related Genes in Synovial Fibroblasts With Rheumatoid Arthritis

**DOI:** 10.3389/fimmu.2021.619392

**Published:** 2021-03-26

**Authors:** Si Wang, Jing Xu, Yuanxu Guo, Yongsong Cai, Xiaoyu Ren, Wenhua Zhu, Manman Geng, Liesu Meng, Congshan Jiang, Shemin Lu

**Affiliations:** ^1^Department of Biochemistry and Molecular Biology, School of Basic Medical Sciences, Institute of Molecular and Translational Medicine (IMTM), Xi’an Jiaotong University Health Science Center, Xi’an, China; ^2^Key Laboratory of Environment and Genes Related to Diseases (Xi’an Jiaotong University), Ministry of Education, Xi’an, China; ^3^Department of Joint Surgery, Xi’an Hong Hui Hospital, Xi’an Jiaotong University Health Science Center, Xi’an, China

**Keywords:** DICER1 gene, apoptosis, proliferation, inflammation, miRNA family, miRNA – microRNA, rheumatoid arthritis

## Abstract

**Objectives:**

Mounting evidence has demonstrated that microRNAs (miRNAs) participate in rheumatoid arthritis (RA). The role of highly conserved miR-15/107 family in RA has not been clarified yet, and hence investigated in this study.

**Methods:**

Reverse transcription-quantitative polymerase chain reaction (RT-qPCR) was used to evaluate the expression of miRNAs and genes. Cell counting kit 8 (CCK-8) and FACS were used to detect proliferation and apoptosis. Protein expression was detected by using Western blotting. mRNA deep sequencing and cytokine antibody array were used to analyze differentially expressed genes, signaling pathways and cytokines.

**Results:**

The expression of miR-15a, miR-103, miR-497, and miR-646 was found decreased, while miR-424 increased in RA patients. MiR-424 and miR-497 were further investigated and the results showed that they could regulate the expression of multiple genes in rheumatoid arthritis synovial fibroblast (RASF) and affect signaling pathways. At the protein level, miR-497 mimic altered all the selected inflammation-related genes while miR-424 inhibitor only affected part of genes. MiR-497 mimic, rather than miR-424 inhibitor, had significant effects on proliferation and apoptosis of RASF. DICER1 was found to positively regulate the expression of miR-424 and miR-497, while DICER1 was also negatively regulated by miR-424. The increase of miR-424 could reduce miR-497 expression, thus forming a loop, which facilitated explaining the dysregulated miR-424 and miR-497 in RA.

**Conclusion:**

The miR-424 and miR-497 of miR-15/107 family affect cell proliferation and apoptosis in RA, and the proposed miR-424-DICER1-miR-497 feedback loop provides a novel insight into regulating miRNA expression and a candidate target for controlling RA.

## Introduction

Rheumatoid arthritis (RA) is a systemic disease characterized by synovial inflammation with unclear etiology. The incidence of RA accounts for about 1% of the world’s total population (1). The onset age of RA is generally between 35-50 years old (2, 3). The inflammation mostly starts from small joints, which are symmetrical and invasive arthritis, often accompanied by extra-articular organs and systems. As the disease progresses, it can lead to joint deformities and malfunction (4).

MicroRNAs (miRNAs) are non-coding small RNAs of approximately 22 nucleotides in length and are highly evolutionarily conserved (5). Existing studies have shown that miRNAs participate in cell proliferation, apoptosis, differentiation, and a series of cell biological behaviors related to the cell cycle (6). In RA, miRNAs are also involved in the pathogenesis of the disease. For instance, miR-155 and miR-146 are up-regulated in RA synovial tissue and participate in the destructive regulation of RASF (7). MiR-34a is down-regulated in RASF and plays a pro-apoptotic role through the apoptotic protein X-linked inhibitor of apoptosis (XIAP) (8). MiR-140 is down-regulated in synovial tissue and can inhibit cell proliferation (9). Although miRNAs research in RA is continuously expanding, some key miRNAs and miRNA families in RA are worth further investigation.

MiR-15/107 gene family contains miR-15a-5p, miR-15b-5p, miR-16-5p, miR-103a-3p, miR-107, miR-195-5p, miR-424-5p, miR-497-5p, miR-503-5p, miR-646 and miR-6838-5p. Members of this family are defined based on the highly conserved seed sequence “AGCAGC” near the 5’ end of the mature miRNA (**Supplementary Table 1**). The regulation of miRNAs in this family affects various cell behaviors such as cell division and metabolism and is also involved in the pathogenesis of various human diseases (10). MiRNAs in this family have been rarely reported in RA, but some of these reports are worthy of reference. For example, some studies suggested that the expression change of miR-16 in serum from RA patients can be used as biomarkers for disease detection (11), while miR-15a can promote apoptosis in the synovium of mice (12), inhibit proliferation of MH7A cell and has a regulatory effect on NF-κB (13). Our previous study analyzed the characteristics of the miR-15/107 family from the perspective of sequence and target genes (14), so this work will explore the role of the miR-15/107 family and the impact of its family members on the expression of inflammation-related genes in RA.

## Materials and Methods

### Patients and Tissue Collection

Human synovial tissue samples were collected from 8 Osteoarthritis (OA) patients and 8 RA patients during knee arthroplasty surgery (Department of Joint Surgery, Xi’an Hong Hui Hospital, Xi’an, China). RASF cells were isolated from synovial tissues of 4 RA patients as previously described (15). This study conformed to the Declaration of Helsinki and was performed with the approval of the human research ethics committee of Xi’an Jiaotong University. All patients fulfilled the American College of Rheumatology criteria for RA classification and written informed consent was obtained from all patients. Information on all patients is summarized in **Supplementary Table 2**.

### Cell Culture

Human synovial sarcoma cell line SW982 and RASF cells were cultured in DMEM (GIBCO life technologies, U.S.A.) medium supplemented with 10 % fetal bovine serum (GIBCO life technologies, U.S.A.), 100 U/ml penicillin and 100 μg/ml streptomycin and incubated at 37°C in humid conditions provided with 5 % CO_2_. RASF cells at the 3rd to 6th generation were used for research.

### Transfection of miRNA Mimic or Inhibitor

Gain or loss of miRNA function was achieved by transfection of 10 nM miRNA mimics or 100 nM inhibitors with Lipofectamine 3000 (Invitrogen, U.S.A.). The sequence information of mimics and inhibitors is shown in **Supplementary Table 3**. SW982 and RASF cells (1.5×10^5^ cells/ml) were seeded in 6-well plates overnight before transfection. After transfection for 48h, RNA and protein were harvested in the same batch during each individual cell experiment.

### SiRNA and Plasmid Vectors

The human DICER1 siRNA product was designed and synthesized by GenePharma Company (China). The sequence information is shown in **Supplementary Table 4**. 10nM siRNA was used for cell transfection in 6-well plates. RNA and protein samples were harvested after 48 h of transfection. 5 μg of the plasmid carrying the full-length CDS of human DICER1 (pCAGGS-hs-Dicer1 from Phil Shap’s Lab, Addgene plasmid #41584 (16)) was transfected into a 6-well plate for DICER1 gene overexpression, plasmid pCAGGS-MCS empty vector was used in control group. Lipofectamine 3000 (Invitrogen, USA) was used for the transfection of siRNA or plasmid vectors. EZNA™ Endo-Free Plasmid Kit (Omega, USA) was introduced to prepare plasmids for cell transfection.

### RNA Extraction and RT-qPCR

Total RNA was isolated by TRIZOL reagent (Invitrogen, U.S.A.) and quantified by NanoDrop 2000 (Thermo Fisher Scientific, U.S.A.). Total RNA for universal cDNA RT reaction was reversely transcribed by oligo d (T) primer from the Transcriptor First Strand cDNA Synthesis Kit (Roche, Mannheim, Germany). MiRNA cDNA from total RNA was synthesized by Mir-x miRNA First-Strand Synthesis Kit (Takara, Japan). Real-time PCR quantification was performed using Mx3000P (Agilent, U.S.A.) and Fast Start Universal SYBR Green Master (Roche, Mannheim, Germany). The expression of genes and miRNAs was normalized by GAPDH and U6. All data were analyzed by the 2^-ΔΔCt^ (semi-quantitative) method. The detailed information of primers was listed in **Supplementary Table 5**. All primers were synthesized by GENEWIZ Company (Suzhou, China).

### Western Blotting

Total protein was collected and isolated with RIPA lysis buffer (Roche, Mannheim, Germany) and then quantified by using BCA kit (Thermo Fisher Scientific, U.S.A.). Twenty μg of each protein sample was loaded and separated by the 8-10 % SDS-PAGE gels, and then transferred to PVDF membranes (Millipore, Billerica, MA, U.S.A.). After blocking with 10 % skim milk for 2 h, the membrane was incubated with antibodies against PDCD4 (CST, 9535T, U.S.A.), BCL2 (Abclonal, A2212, China), MMP3 (Abclonal, A2426, China), MMP13 (Proteintech, 18165-I-AP, U.S.A.), CCND1 (CST, 2922S, U.S.A.), CCND3 (CST, 2936T, U.S.A.), CCNE1(Proteintech, 11554-I-AP, U.S.A.), CCNE2 (Proteintech, 11935-I-AP, U.S.A.) and GAPDH (Proteintech, U.S.A.). ECL kit (Millipore, WBKLS0050, U.S.A.) was used for chemiluminescence. The bands were captured and scanned using Syngene Image system and Genesys software.

### Cell Apoptosis Assay

Apoptotic cells were determined by Annexin V FITC Apoptosis Detection Kit I (BD Pharmingen, U.S.A.). After transfection with 100 nM miR-424 inhibitor and 10 nM miR-497 mimic for 24h, RASF cells were collected in 1.5 ml tubes. After washing the cells with 1 ml PBS, 1×binding buffer was added into each tube, then a mixture of Annexin V-FITC and PI was added, and the tubes were kept at room temperature in a dark place for 15 min. Next, 1×binding buffer was added into tubes and mixed thoroughly. The cell suspension was loaded for apoptosis detection, which was conducted via the Guava FACS machine (Millipore, U.S.A.) and the software Guava2.2.2.

### Cell Counting Kit-8 Assay

RASF was transfected with 100 nM miR-424 inhibitor, 10 nM miR-497 mimic, or 10 nM negative control (NC). Every well in a 96-well plate was inoculated with approximately 2000 cells. After 3 days of incubation, 10 μl of CCK-8 (Beyotime Biotechnology, China) was added to each well for 1h incubation. The optical density (OD) value was obtained by the multiskan spectrum (Thermo, U.S.A.) at a wavelength of 450 nm. The fold change of optical density (OD) value from three independent cell experiments was used for data analysis.

### Cytokine Expression Profiling Assay

MiRNA mimics were transfected in RASF, and the medium was changed to DMEM with 0.2 % FBS after 4 h transfection. After 48 h transfection, the medium was collected and centrifuged at 2000 rpm for 10 min at 4°C, and the supernatant was transferred to a new tube and stored in aliquots at -80°C before assay. The RASF cells used for the cytokine array were isolated from the synovial tissues of 4 RA patients. After transfection with corresponding miRNA mimics and controls, the cell supernatant was collected and analyzed using RayBio® Human Cytokine Antibody Array C5.The array detects 80 human cytokines in conditioned cell culture media. The arrangement of cytokines was shown in **Supplementary Table 6**. Both the array detection and analysis were performed by RayBiotech Company.

### MRNA Deep Sequencing by Illumina HiSeq

After transfected with miR-424 or miR-497 mimics, the total RNA of each sample from RASF was extracted by TRIzol Reagent (Invitrogen, U.S.A.) and qualified by NanoDrop (Thermo Fisher Scientific, U.S.A.). Total RNA of 1.5 μg with RIN value above 7 was used for the following library preparation. NC-1 corresponds to 424-1 and 497-1 as one patient sample in total 4 patients. Next-generation sequencing library was constructed according to the manufacturer’s protocol (NEBNext^®^ Ultra™ RNA Library Prep Kit for Illumina^®^). The detection and analysis of mRNA sequencing were performed by Genewiz Company. The entire dataset was deposited in the GEO database (GSE159618).

### Statistical Analysis

Experimental data were expressed as mean ± standard error of the mean (SEM). Differences between the groups were analyzed using the Mann-Whitney test. *p* < 0.05 was considered statistically significant (***p < 0.001, ** *p* < 0.01, * *p* < 0.05).

## Results

### Expression of miR-15/107 Family Members in Patient Tissues and Expression of Inflammation-Related Genes After Up-Regulation of miR-15a, miR-103, miR-424, miR-497, and miR-646

**Figure 1:**
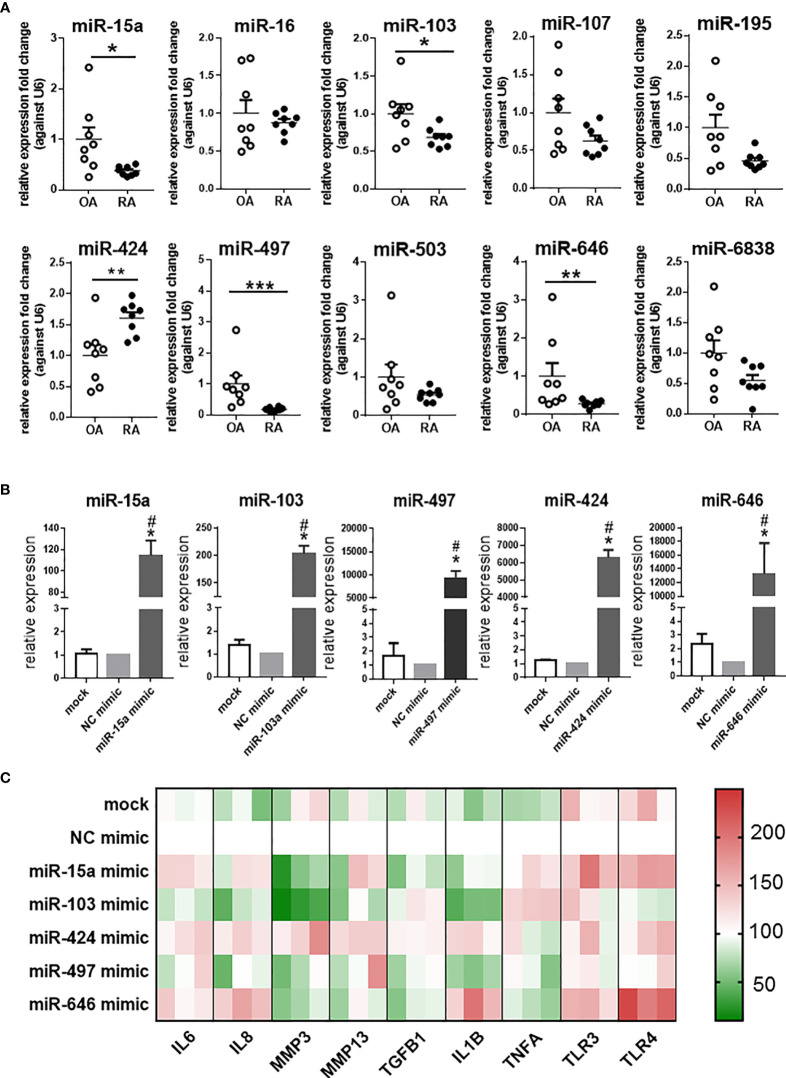
The expression of miR-15/107 family in RA and OA synovial tissues and pro-inflammatory cytokines, MMPs, Toll-like receptors in SW982 cells. **(A)** The expression of 10 mature miR-15/107 family members in human synovial tissue form RA or OA patients determined by using RT-qPCR. **(B)** The expression of 5 miRNAs after transfected with 10 nM mimics determined by using RT-qPCR. **(C)** The expression of pro-inflammatory cytokines (*IL1B, IL6, IL8, TNFA, TGFB1*), MMPs (*MMP3, MMP13*) and Toll-like receptors (*TLR3, TLR4*) determined by RT-qPCR and the results were shown through heatmap, each color block in the heatmap represents an independent cell experiment. Red, green, and white signals indicate relatively high, low, and baseline (mock group) expression levels. **(A)** Bar represents mean ± SEM of samples from OA and RA patients (both n = 8). **(B, C)**: Bar represents mean ± SEM from three independent cell experiments in SW982 cell. Triplicates were used for each transfection and RT-qPCR experiment. Mann-Whitney U test (***P< 0.001, **P< 0.01, *P< 0.05), *: vs. NC mimic, #: vs. mock.

The expression of 10 miRNAs in the miR-15/107 family was detected in synovial tissues from OA and RA patients (**Figure 1A**). The results showed that miR-15a, miR-103, miR-497, and miR-646 were significantly down-regulated in RA patients than in the OA patients, while the miR-424 expression was up-regulated. Therefore, in the following experiments, we focused on these five miRNAs with altered expression and explored their roles in RA.

In SW982 cell line, we transfected the 10 nM mimic of miR-15a, miR-103, miR-497, miR-646, and miR-424. As shown in **Figure 1B**, the expression of these miRNAs increased after transfected with miRNA mimics. Based on these results, we further tested the expression of several inflammation-related genes (*IL1B*, *IL6*, *IL8*, *MMP3*, *MMP13*, *TGFB1*, *TNFA*, *TLR3*, *TLR4*). In miR-15a mimic-treated cells, the expression of *MMP3*, *TGFB1* and *IL1B* significantly decreased, other genes, such as *IL6*, *TLR3* and *TLR4* increased. The expression of most genes (*IL1B*, *IL6*, *IL8*, *MMP3*, and *TLR4*) in miR-103 mimic-treated cell displayed down-regulation, but only *TNFA* was up-regulated. The expression of inflammation-related genes in miR-424 mimic-treated cells all increased except *TLR3* and *TNFA*, while in miR-646 mimic-treated cells, the expression of *MMP3*, *MMP13*, *TNFA* and *TGFB1* was down-regulated, and other inflammation-related genes, like *IL6*, *IL8*, *IL1B* and *TLR4* were up-regulated. In miR-497 mimic-treated cells, the expression of other inflammation-related genes was not significantly different except that the expression of *IL1B* was down-regulated. Summarizing these results as a heatmap (**Figure 1C**), it was shown that after transfected with miR-424 mimic, the expression of inflammation-related genes in cells exhibited opposite states compared with miR-103 and miR-497 mimic-treated cells. The results further demonstrated that the expression of miR-424 and miR-497 in RA has the opposite effect on the regulation of inflammation-related genes, so it will be more representative to explore their roles.

### mRNA Deep Sequencing Analysis of Differentially Expressed Genes and Signaling Pathways in miR-424 and miR-497 Mimic-Treated RASF

**Figure 2:**
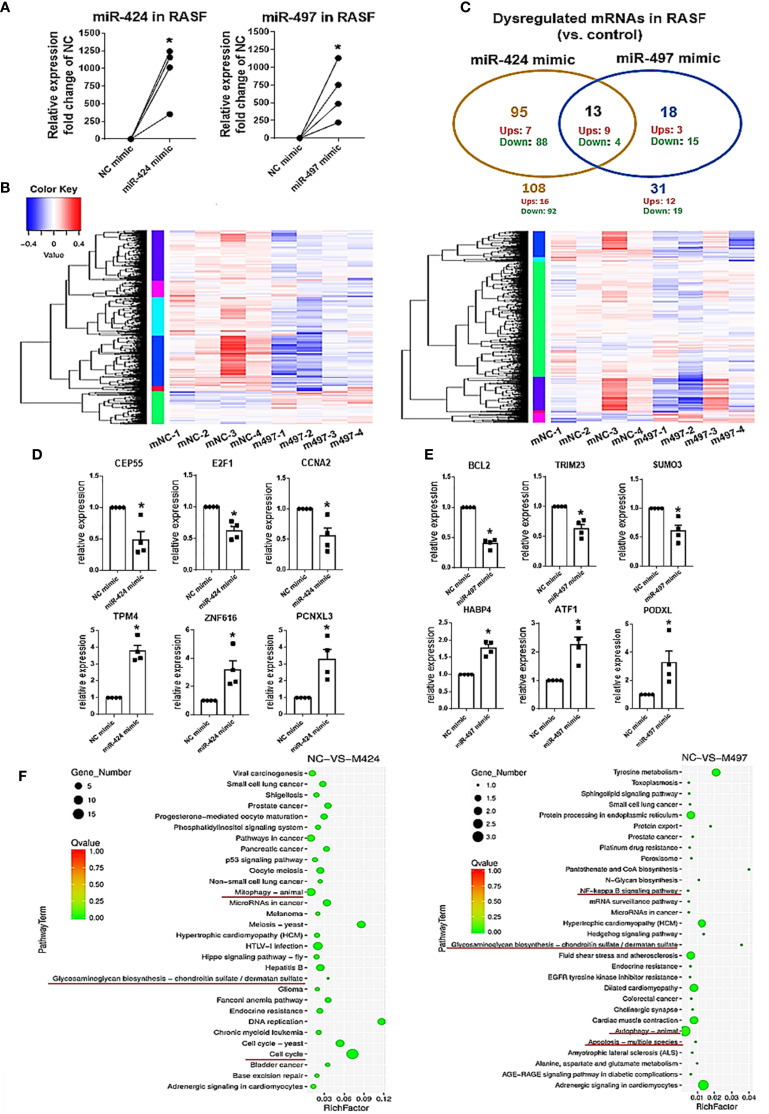
MRNA deep sequencing results and validation after gain of miR-424 and miR-497 function for 48 h in RASF. **(A)** The expression of miR-424 and miR-497 in RASF transfected with 10 nM mimic 48h detected by using RT-qPCR. **(B)** Heat map of all dysregulated genes from mRNA deep sequencing from 10nM miR-424 or miR-497 mimic-treated RASF cells. **(C)** Venn diagram of dysregulated genes in miR-424 and miR-497 mimic-treated cell from miRNA deep sequencing results. **(D, E)** RT-qPCR results of dysregulated genes in miR-424 mimic-treated cells **(D)** and miR-497 mimic-treated cells **(E)**. **(F)** KEGG signal pathway analysis diagram. RASF cells isolated from synovial tissue of 4 RA patients were transfected with miR-424 or miR-497 mimics and inhibitors, respectively. Triplicates were used for each transfection experiment. Bar: mean ± SEM of samples from 4 patients. *P*<0.05, *: vs. NC mimic, using Mann-Whitney U test.

We used mRNA deep sequencing to explore the role of miR-424 and miR-497 further. RASF was isolated from the synovial samples of 4 RA patients, and we established the cell with up-regulated miRNA expression for sequencing detection by using miR-424 and miR-497 mimics (**Figure 2A**). The heatmaps were performed to show the expression changes of 525 genes in miR-424 and miR-497 mimic-treated cells (**Figure 2B**). We screened genes with differential expression fold change ≥ 1.5 and FDR ≤ 0.05. 108 genes were dysregulated in the miR-424 mimic-treated group, of which 16 genes were up-regulated, and the rest were down-regulated. 31 genes were dysregulated in the miR-497 mimic treatment group, and 12 genes were up-regulated. They shared 13 genes in common, 9 of which were up-regulated (**Figure 2C**). Combined with literature reports (**Supplementary Tables 7**, **8**), we selected some genes related to RASF for verification. In miR-424 mimic-treated RASF, we chose three genes to detect and found that the mRNA expression of *CEP55, E2F1* and *CCNA2* decreased (**Figure 2D**) while in miR-497 mimic-treated RASF, the mRNA expression of *BCL2, TRIM23*, and *SUMO3* also reduced (**Figure 2E**). Among the up-regulated genes, we selected PCNXL3, TPM4, and ZNF616 in miR-424 mimic-treated group for verification, and ATF1, HABP4, PODXL in the miR-497 mimic-treated group. The results showed that the expression of these genes was both up-regulated (**Figure 2E**). The validation results (**Figure 2E**) of these representative genes were all consistent with mRNA deep sequencing results.

In KEGG pathway analysis, we found that autophagy, cell cycle, and glycosaminoglycan biosynthesis signal pathways changed after miR-424 mimic transfection. The NF-κB and glycosaminoglycan biosynthesis pathway changes after miR-497 mimic transfection suggest that inflammatory and joint synovium destruction were regulated (**Figure 2F**). Taken together, these results indicate that miR-424 and miR-497 may play a role in cell proliferation and apoptosis, and were closely related to the degradation of extracellular matrix.

### Effects of miR-424 and miR-497 on the Genes Involved in Cell Survival and Matrix Degradation

**Figure 3:**
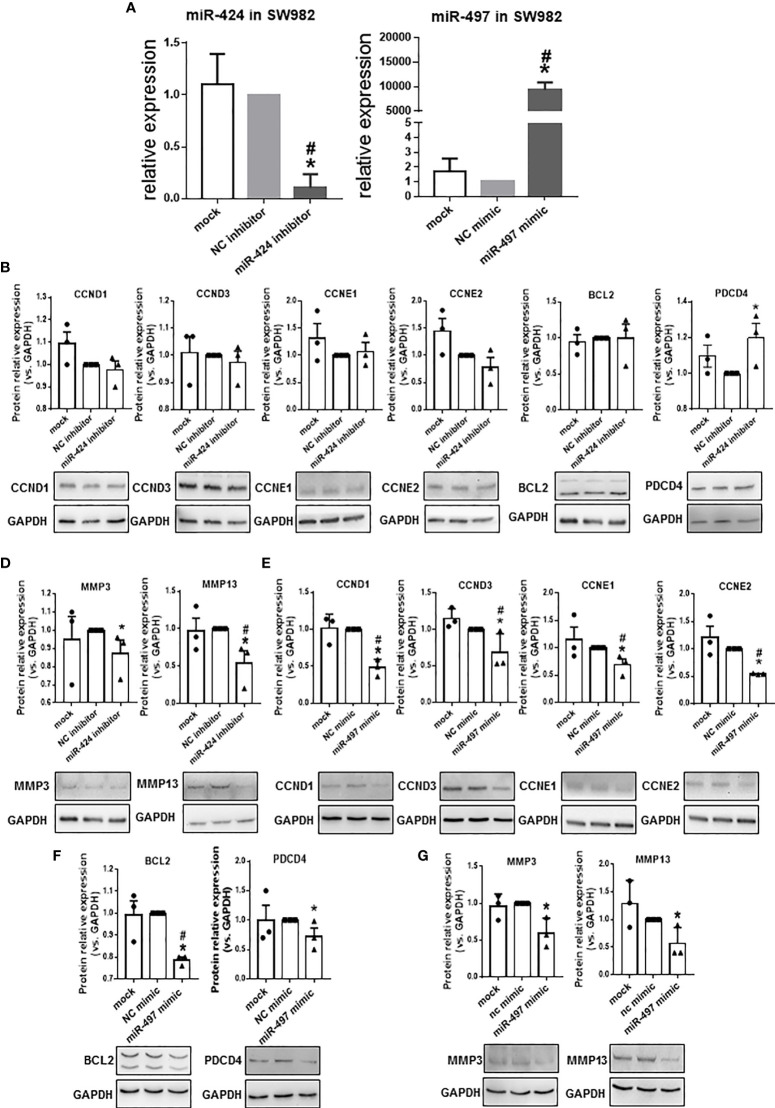
Expression of proliferation, apoptosis, and extracellular matrix degradation related genes at protein level in SW982 cells. **(A)** The expression of miR-424 and miR-497 determined by using RT-qPCR from SW982 cell transfected with 100 nM miR-424 inhibitor or 10 nM miR-497 mimic for 48h. **(B–D)** Western blotting results of CCND1, CCND3, CCNE1, CCNE2 **(B)**, BCL2 and PDCD4 (**C**), MMP3, and MMP13 **(D)** in 100 nM miR-424 inhibitor-treated cells. **(E–G)** Western blotting result of CCND1, CCND3, CCNE1, CCNE3 **(E)**, BCL2 and PDCD4 **(F)**, MMP3 and MMP13 **(G)** in 10 nM miR-497 mimic-treated cells. Bar: mean ± SEM from three independent cell experiments in SW982 cell. Triplicates were used for each transfection and RT-qPCR experiment. *P*<0.05, *: vs. NC, #: vs. mock using Mann-Whitney test.

Since the expression of miR-424 was elevated, and the expression of miR-497 decreased in RA patients’ synovium, from the perspective of potential disease treatment, we performed several experiments in miR-424 inhibitor and miR-497 mimic-treated cells. RT-qPCR results confirmed the successful reduction of miR-424 and increase of miR-497 in SW982 cells transfected with miR-424 inhibitor and miR-497 mimic, respectively (**Figure 3A**). Combining the results in the TARBASE database (**Supplementary Tables 9**, **10**), the target genes, including CCND1, CCND3, CCNE1, and CCNE2, were further selected as the cell proliferation-related genes, PDCD4 and BCL2 were selected as the apoptosis-related genes. Besides, MMP3 and MMP13 were chosen as the extracellular matrix degradation-related genes. We tested the expression of these genes at the protein level. In cells treated with miR-424 inhibitor, the expression of most genes was unchanged, except for the down-regulation of MMP3 and MMP13, and the up-regulation of PDCD4 (**Figures 3B–D**). After transfected with miR-497 mimic, the expression of CCND1, CCND3, CCNE1, CCNE2, PDCD4 and BCL2 significantly decreased (**Figures 3E, F****)**. Meanwhile, the expression of MMP3 and MMP13 also decreased (**Figure 3G**). These results demonstrated that miR-497 mimic has significant effects on genes related to cell proliferation, apoptosis, and extracellular matrix degradation, while miR-424 inhibitor also has effects on genes related to apoptosis and extracellular matrix degradation. Compared with miR-424, the effect of miR-497 seemed more promising.

### Effects of miR-424 and miR-497 on the Proliferation and Apoptosis of RASF

**Figure 4:**
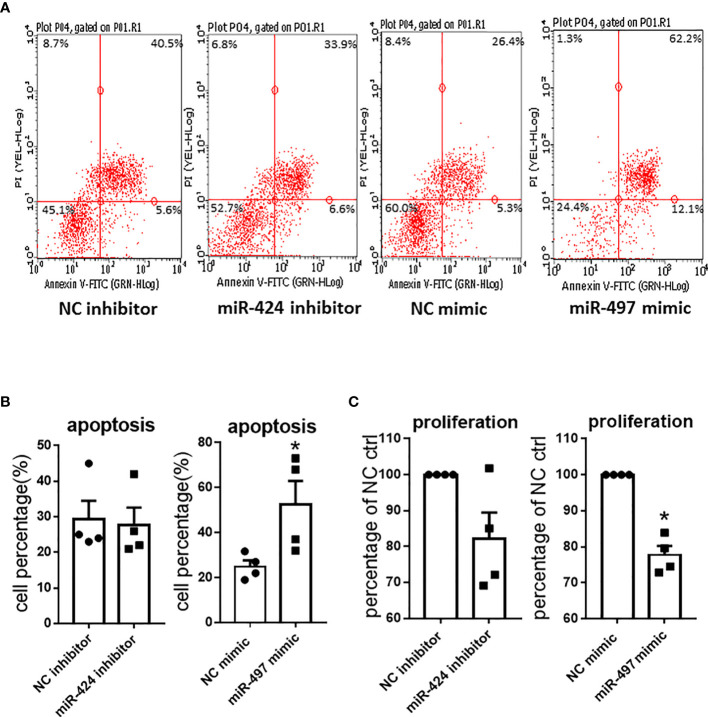
Apoptosis and proliferation result caused by gain or loss of miR-424 and miR-497 function in RASF. **(A)** Flow cytometry representative images of Annexin V-FITC and PI double staining after transfected with 100 nM miR-424 inhibitor and 10 nM miR-497 mimic for 24 h. Four quadrants represent viable cells; early apoptotic cells, late apoptotic cells and dead cells. **(B)** Percentage of apoptotic cells in transfected cells. **(C)** Proliferation result detected by cell counting kit-8 assay (CCK8) after transfected with 100 nM miR-424 inhibitor and 10 nM miR-497 mimic for 72h. RASF cells isolated from synovial tissue of 4 RA patients were transfected with miR-424 inhibitor or miR-497 mimic, respectively. Bar: mean ± SEM of samples from 4 patients. *: *P* < 0.05 using the Mann-Whitney U test.

We further investigated the effects of miR-424 inhibitor and miR-497 mimic on cell proliferation and apoptosis in RASF. The Annexin V-FITC and PI double staining was carried out to determine the effects on apoptosis. CCK8 was performed to detect RASF proliferation. According to the scatter plot (**Figure 4A**), after transfected with miR-497 mimic, the apoptotic ratio significantly increased compared with the NC group. However, there was no change of apoptotic ratio after RASF was transfected with miR-424 inhibitor (**Figure 4B**). In miR-497 mimic-treated RASF, the cell proliferation decreased, while in miR-424 inhibitor-treated RASF, proliferation was not significantly changed compared with NC group (**Figure 4C**).

### Analysis of Differentially Expressed Cytokines in Cell Supernatant

**Figure 5:**
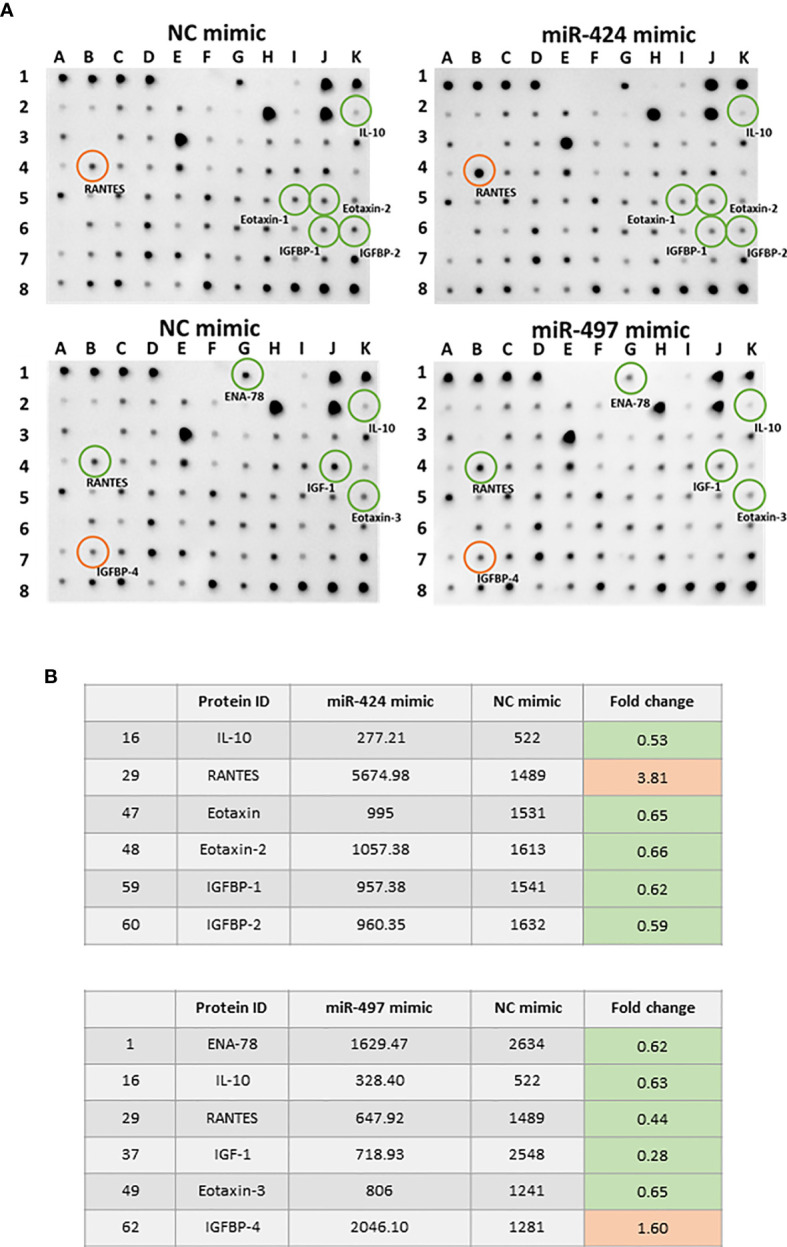
Cytokine expression alteration caused by gain of miR-424 and miR-497 function for 48 h in RASF. **(A)** Image result of cytokine array from the supernatant of RASF transfected with 10 nM miR-424 or miR-497 mimic for 48 h. Red circle: Elevated expression. Green circle: Reduced expression. **(B)** All differentially expressed cytokines in miR-424 and miR-497 with fold change ≤0.67 or fold change≥1.5. RASF cells isolated from synovial tissue of 4 RA patients were transfected with miR-424 and miR-497 mimics. Triplicates were used for each transfection experiment.

We performed a cytokines array assay to detect the cytokines secreted in RASF supernatants. Comparing with the NC group, 10 out of 80 detected cytokines showed significant differences after miR-424 or miR-497 mimic treatment (**Figure 5A**). The cytokines in **Figure 5B** were quantitative data of miR-424 and miR-497 with fold change≥1.5 and≤0.67. Comparing the expression of these cytokines, the protein secreted by RASF showed opposite regulation. In miR-424 mimic-treated cell, the cytokines changed significantly were IL-10, RANTES, Eotaxin, Eotaxin-2, IGFBP-1 and IGFBP-2, among which RANTES (CCL5) increased most obviously. In miR-497 mimic-treated cells, ENA-78, IL-10, RANTES, IGF-1, eotaxin-3 and IGFBP-4 have apparent changes. It is worth noting that the expression of RANTES was quite different in the supernatant of two miRNA-treated cells. The secretion of RANTES increased in miR-424 mimic-treated cell, while in miR-497 mimic-treated cell, the secretion of RANTES decreased. The expression of IGPBP4 and ENA78 also showed the opposite state. These results indicated that miR-424 and miR-497 have opposite effects on the secretion of certain inflammation-related cytokines.

### Interaction of miR-424 and miR-497 Through DICER1

**Figure 6:**
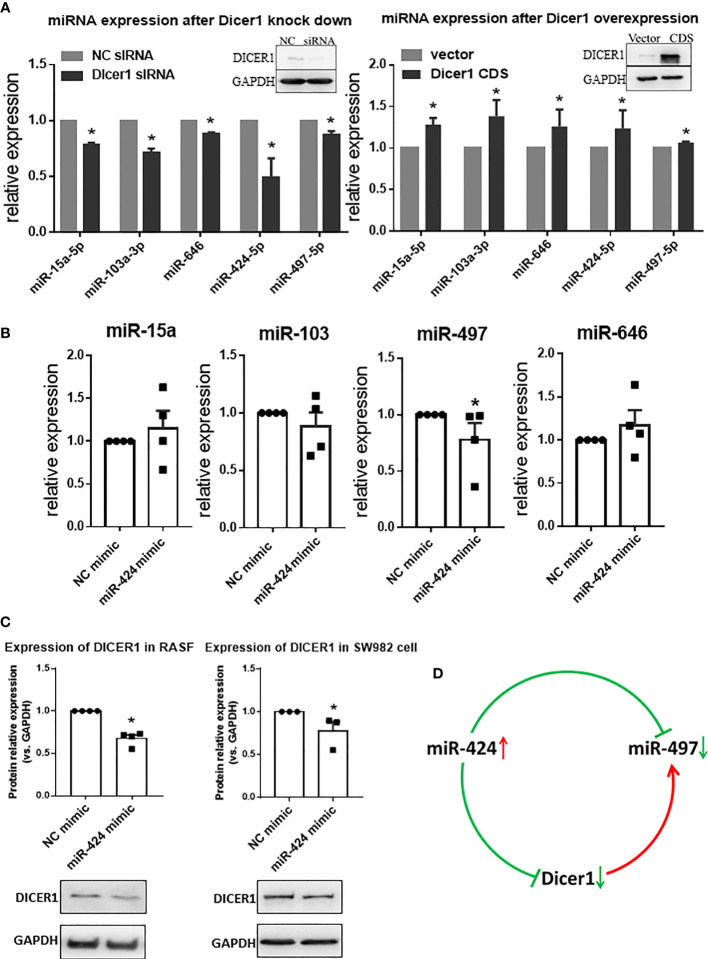
Interaction of miR-424 and miR-497 with DICER1. **(A)** The expression of miR-15a, miR-103a, miR-424, miR-497 and miR-646 during loss and gain of DICER1 function in SW982 cells detected by RT-qPCR. Loss and gain of DICER1 function were validated by western blotting, representative image shown. **(B)** The expression of miR-15a, miR-103, miR-497 and miR-646 after transfected with 10 nM miR-424 mimic for 48 h by RT-qPCR in RASF. **(C)** Western blotting results of DICER1 after transfected with 10 nM miR-424 mimic for 48 h in RASF and SW982 cells. **(D)** Brief mechanism diagram of negative feedback loop. RASF cells were isolated from synovial tissue of 4 RA patients. Bar: mean ±SEM from 4 patients in RASF and mean ± SEM from 3 independent cell experiments in SW982 cell. Triplicates were used for each transfection and RT-qPCR experiment. *: *P*<0.05 compared with NC.

The opposite expression of miR-424 and miR-497 in RA synovium brought out the question that other genes may be involved in regulation between them. As an essential gene for miRNA maturation and a target gene for miRNA, we focus on DICER1. The targeting relationship among DICER1 and miR-424 and miR-497 has been verified (**Supplementary Table 11**). The effect of DICER1 on miRNAs was determined, and the results showed that when DICER1 was knocked down in SW982 cell line, the expression of miR-15a, miR-103, miR-424, miR-497 and miR-646 was reduced, while DICER1 overexpression could increase the expression of these miRNAs (**Figure 6A**). Because miR-424 is the unique miRNA with increased expression in RA synovium, we detected the expression of miR-15a, miR-103, miR-497 and miR-646 in RASF transfected with miR-424 mimic. The results showed that only miR-497 was down-regulated, while the other 4 miRNAs were not significantly changed (**Figure 6B**). This result indicated that there might be a regulatory relationship between miR-424 and miR-497. In addition, the protein expression of DICER1 could be down-regulated by miR-424 mimic (**Figure 6C**) in both RASF and SW982 cell lines. Combining these results, we propose a mechanism that the increase of miR-424 reduces the expression of miR-497 through a miR-424-DICER1-miR-497 feedback loop, resulting in the opposite miR-424 and miR-497 expression in RA synovium (**Figure 6D**).

## Discussion

In the present study, we have mainly focused on the role of the miR-15/107 family in RA and found that miR-424 and miR-497 are reversely expressed in RA. At the same time, miR-424 and miR-497 had the opposite capacity to regulate some inflammation-related genes and inflammatory cytokines. Besides, miR-497 inhibited proliferation and promoted apoptosis in RASF cells. A miR-424-DICER1-miR-497 feedback loop has been further considered as a possible underlying mechanism of increased miR-424 and reduced miR-497 expression from the same miRNA family in RA synovial tissues.

The expression of inflammation-related genes and cytokines is closely related to the occurrence and development of RA. The release of IL-6 and IL-1 can cause inflammation of the synovium (17). The toll-like receptors such as TLR2, TLR3, TLR4, TLR5, TLR6, TLR-7, and TLR9 are overexpressed in RASF and participate in RA (18, 19). The expression of MMP3 and MMP13 is considered as a crucial marker of RA disease activity (20–22). In our results, miR-424 and miR-497 had opposite expression levels in RA patient samples, and the increased expression of miR-424 and miR-497 caused inflammation-related genes to show an opposite expression trends in SW982 cells. The cytokine array results also reflect the same regulatory effect. RANTES mediates T cells in RA and other cells involved in inflammation (23), while RANTES can also induce MMP1 and MMP13 expression in human RASF (24). The overexpression of RANTES in RASF is a potential mediator that affects the intensity and composition of cell infiltration in the joints of inflammatory arthritis (25). Therefore, the different expression levels of RANTES and other cytokines further confirmed that miR-424 and miR-497 have different roles in regulating cytokines secretion.

We screened three genes related to miR-497 mimic (BCL2, TRIM23, SUMO3) based on the mRNA deep sequencing results. Previous research showed that the high transcription level of BCL2 in SF Terg cells could suppress cell apoptosis (26). In the deep sequencing results, up-regulating miR-497 reduced the expression of BCL2 at mRNA levels. Western blotting data also demonstrated that miR-497 mimic could significantly reduce the expression of BCL2 at the protein level. These results suggest that miR-497 mimic may play a role in promoting apoptosis. This speculation is consistent with the results of the cell proliferation and apoptosis detection, so we assume that miR-497 mimic can promote the apoptosis of RASF cells. The expression of SUMO3 and TRIM23 in the sequencing results is also reduced, but in a report, the high expression of TRIM23 and the knockdown of SUMO3 could promote the activation of NF-κB pathway (27, 28), which means that the effect of miR-497 is not unidirectional, but it does effect the inflammatory signaling pathways and cell apoptosis.

Besides RA, miR-424 and miR-497 also show opposite expression trends in other disease. In middle cerebral artery occlusion (MCAO) mouse models, the expression of miR-424 significantly reduced (29), while the expression of miR-497 increased (30). In squamous cell carcinoma of skin, miR-424 expression increased, and miR-497 expression decreased (31). In colorectal cancer, the expression of miR-424 and miR-497 co-target IGF1-R and are both down-regulated, but only the up-regulated expression of miR-497 will result in the inhibition of IGF1-R 3’UTR activity (32). To further explain why they have the opposite expression tendency in RA patient samples, we focus on DICER1. In RA, the decrease of DICER1 expression aggravates the symptoms in the mouse model of joint inflammation (33), and the lack of DICER1 causes the decrease of RA-related miRNA expression (34). In experimental serum metastatic arthritis established with DICER1-deficient mice, the imbalance of miRNA biosynthesis associated with enhanced inflammatory response (35), which confirms the vital role of DICER1 in RA. As a target gene, it has been shown that exosomes secreting gastric cancer can transfer miR-107 to the host myeloid-derived suppressor cells, where miR-107 induces MDSC amplification and activation by targeting DICER1 (36). The bidirectional effect of DICER1 is the key factor for it to become an intermediate molecule. Here, we propose a mechanism that the increase of miR-424 reduces the expression of miR-497 through a miR-424-DICER1-miR-497 feedback loop, leading to the opposite miR-424 and miR-497 expression in RA synovium. The feedback loop of DICER1 is not necessarily the only hidden clue, however, our results provide a novel insight into understanding miRNA expression and new candidate targets for controlling RA.

## Data Availability Statement

The datasets presented in this study can be found in online repositories. The names of the repository/repositories and accession number(s) can be found below: https://www.ncbi.nlm.nih.gov/geo/query/acc.cgi?acc=GSE159618.

## Ethics Statement

The studies involving human participants were reviewed and approved by human research ethics committee of Xi’an Jiao tong University. The patients/participants provided their written informed consent to participate in this study.

## Author Contributions

Study design: CJ and SL. Data collection and analysis: SW, CJ, YG, and JX. Contribution of new reagents or analytical tools: WZ, MG, and LM. Manuscript preparation: SW, CJ, YG, JX. Isolation of patient’s primary cells: YC and XR. Financial support: CJ and SL. Finalized the manuscript: SW, CJ, and SL. All authors contributed to the article and approved the submitted version.

## Funding

This work is supported by the National Science Foundation of China (grant No. 81671629, 81701619, 81970029, and 81902249) and Shaanxi Province Natural Science Foundation (Project No. 2018JM7057).

## Conflict of Interest

The authors declare that the research was conducted in the absence of any commercial or financial relationships that could be construed as a potential conflict of interest.

## References

[1] Alamanos Y Drosos AA. Epidemiology of adult rheumatoid arthritis. Autoimmun Rev (2005) 4:130–6. 10.1016/j.autrev.2004.09.002 15823498

[2] Ahlmen M Svensson B Albertsson K Forslind K Hafstrom I Group BS. Influence of gender on assessments of disease activity and function in early rheumatoid arthritis in relation to radiographic joint damage. Ann Rheum Dis (2010) 69:230–3. 10.1136/ard.2008.102244 19158113

[3] Areskoug-Josefsson K Oberg U. A literature review of the sexual health of women with rheumatoid arthritis. Musculoskeletal Care (2009) 7:219–26. 10.1002/msc.152 19242923

[4] Nakamachi Y Kawano S Takenokuchi M Nishimura K Sakai Y Chin T. MicroRNA-124a is a key regulator of proliferation and monocyte chemoattractant protein 1 secretion in fibroblast-like synoviocytes from patients with rheumatoid arthritis. Arthritis Rheum (2009) 60:1294–304. 10.1002/art.24475 19404929

[5] Bartel DP. MicroRNAs: genomics, biogenesis, mechanism, and function. Cell (2004) 116:281–97. 10.1016/S0092-8674(04)00045-5 14744438

[6] Huang Y Shen XJ Zou Q Zhao QL. Biological functions of microRNAs. Bioorg Khim (2010) 36:747–52. 10.1134/S1068162010060026 21317939

[7] Stanczyk J Pedrioli DM Brentano F Sanchez-Pernaute O Kolling C Gay RE. Altered expression of MicroRNA in synovial fibroblasts and synovial tissue in rheumatoid arthritis. Arthritis Rheum (2008) 58:1001–9. 10.1002/art.23386 18383392

[8] Niederer F Trenkmann M Ospelt C Karouzakis E Neidhart M Stanczyk J. Down-regulation of microRNA-34a* in rheumatoid arthritis synovial fibroblasts promotes apoptosis resistance. Arthritis Rheum (2012) 64:1771–9. 10.1002/art.34334 22161761

[9] Li H Guan SB Lu Y Wang F. MiR-140-5p inhibits synovial fibroblasts proliferation and inflammatory cytokines secretion through targeting TLR4. Biomed Pharmacother = Biomede Parmacother (2017) 96:208–14. 10.1016/j.biopha.2017.09.079 28987944

[10] Finnerty JR Wang WX Hebert SS Wilfred BR Mao G Nelson PT. The miR-15/107 group of microRNA genes: evolutionary biology, cellular functions, and roles in human diseases. J Mol Biol (2010) 402:491–509. 10.1016/j.jmb.2010.07.051 20678503 PMC2978331

[11] Dunaeva M Blom J Thurlings R Pruijn GJM. Circulating serum miR-223-3p and miR-16-5p as possible biomarkers of early rheumatoid arthritis. Clin Exp Immunol (2018) 193:376–85. 10.1111/cei.13156 PMC6149957 29892977

[12] Nagata Y Nakasa T Mochizuki Y Ishikawa M Miyaki S Shibuya H. Induction of apoptosis in the synovium of mice with autoantibody-mediated arthritis by the intraarticular injection of double-stranded MicroRNA-15a. Arthritis Rheum (2009) 60:2677–83. 10.1002/art.24762 19714650

[13] Li G Qiu Z. Deletion of miR-15 Protects Against Rheumatoid Arthritis via Deregulating its Target Gene BCL2L2 and Repressing NF-kappaB Pathway. Ann Clin Lab Sci (2019) 49:581–9. 31611200

[14] Wang S Zhu W Xu J Guo Y Yan J Meng L. Interpreting the MicroRNA-15/107 family: interaction identification by combining network based and experiment supported approach. BMC Med Genet (2019) 20:96. 10.1186/s12881-019-0824-9 31151434 PMC6544937

[15] Cai Y Jiang C Zhu J Xu K Ren X Xu L. miR-449a inhibits cell proliferation, migration, and inflammation by regulating high-mobility group box protein 1 and forms a mutual inhibition loop with Yin Yang 1 in rheumatoid arthritis fibroblast-like synoviocytes. Arthritis Res Ther (2019) 21:134. 10.1186/s13075-019-1920-0 31159863 PMC6547523

[16] Gurtan AM Lu V Bhutkar A Sharp PA. In vivo structure-function analysis of human Dicer reveals directional processing of precursor miRNAs. Rna (2012) 18:1116–22. 10.1261/rna.032680.112 PMC3358635 22546613

[17] Choy E. Understanding the dynamics: pathways involved in the pathogenesis of rheumatoid arthritis. Rheumatol (Oxford) (2012) 51(Suppl 5):v3–11. 10.1093/rheumatology/kes113 22718924

[18] Brentano F Kyburz D Schorr O Gay R Gay S. The role of Toll-like receptor signalling in the pathogenesis of arthritis. Cell Immunol (2005) 233:90–6. 10.1016/j.cellimm.2005.04.018 15963480

[19] Cho ML Ju JH Kim HR Oh HJ Kang CM Jhun JY. Toll-like receptor 2 ligand mediates the upregulation of angiogenic factor, vascular endothelial growth factor and interleukin-8/CXCL8 in human rheumatoid synovial fibroblasts. Immunol Lett (2007) 108:121–8. 10.1016/j.imlet.2006.11.005 17182109

[20] Lerner A Neidhofer S Reuter S Matthias T. MMP3 is a reliable marker for disease activity, radiological monitoring, disease outcome predictability, and therapeutic response in rheumatoid arthritis. Best Pract Res Clin Rheumatol (2018) 32:550–62. 10.1016/j.berh.2019.01.006 31174824

[21] Jungel A Ospelt C Lesch M Thiel M Sunyer T Schorr O. Effect of the oral application of a highly selective MMP-13 inhibitor in three different animal models of rheumatoid arthritis. Ann Rheum Dis (2010) 69:898–902. 10.1136/ard.2008.106021 19497915 PMC2925150

[22] Shervington L Darekar A Shaikh M Mathews R Shervington A. Identifying Reliable Diagnostic/Predictive Biomarkers for Rheumatoid Arthritis. Biomark Insights (2018) 13:1177271918801005. 10.1177/1177271918801005 30262983 PMC6153528

[23] Luterek-Puszynska K Malinowski D Paradowska-Gorycka A Safranow K Pawlik A. CD28, CTLA-4 and CCL5 gene polymorphisms in patients with rheumatoid arthritis. Clin Rheumatol (2017) 36:1129–35. 10.1007/s10067-016-3496-2 27988812

[24] Agere SA Akhtar N Watson JM Ahmed S. RANTES/CCL5 Induces Collagen Degradation by Activating MMP-1 and MMP-13 Expression in Human Rheumatoid Arthritis Synovial Fibroblasts. Front Immunol (2017) 8:1341. 10.3389/fimmu.2017.01341 29093715 PMC5651228

[25] Stanczyk J Kowalski ML Grzegorczyk J Szkudlinska B Jarzebska M Marciniak M. RANTES and chemotactic activity in synovial fluids from patients with rheumatoid arthritis and osteoarthritis. Mediators Inflammation (2005) 2005:343–8. 10.1155/MI.2005.343 PMC1533897 16489254

[26] van der Geest KS Smigielska-Czepiel K Park JA Abdulahad WH Kim HW Kroesen BJ. SF Treg cells transcribing high levels of Bcl-2 and microRNA-21 demonstrate limited apoptosis in RA. Rheumatol (Oxford) (2015) 54:950–8. 10.1093/rheumatology/keu407 25339644

[27] Poole E Groves I MacDonald A Pang Y Alcami A Sinclair J. Identification of TRIM23 as a cofactor involved in the regulation of NF-kappaB by human cytomegalovirus. J Virol (2009) 83:3581–90. 10.1128/JVI.02072-08 PMC2663253 19176615

[28] Frank S Peters MA Wehmeyer C Strietholt S Koers-Wunrau C Bertrand J. Regulation of matrixmetalloproteinase-3 and matrixmetalloproteinase-13 by SUMO-2/3 through the transcription factor NF-kappaB. Ann Rheum Dis (2013) 72:1874–81. 10.1136/annrheumdis-2012-202080 23417988

[29] Zhao H Wang J Gao L Wang R Liu X Gao Z. MiRNA-424 protects against permanent focal cerebral ischemia injury in mice involving suppressing microglia activation. Stroke (2013) 44:1706–13. 10.1161/STROKEAHA.111.000504 23613494

[30] Koutsis G Siasos G Spengos K. The emerging role of microRNA in stroke. Curr Top Med Chem (2013) 13:1573–88. 10.2174/15680266113139990106 23745809

[31] Mizrahi A Barzilai A Gur-Wahnon D Ben-Dov IZ Glassberg S Meningher T. Alterations of microRNAs throughout the malignant evolution of cutaneous squamous cell carcinoma: the role of miR-497 in epithelial to mesenchymal transition of keratinocytes. Oncogene (2018) 37:218–30. 10.1038/onc.2017.315 28925390

[32] Guo ST Jiang CC Wang GP Li YP Wang CY Guo XY. MicroRNA-497 targets insulin-like growth factor 1 receptor and has a tumour suppressive role in human colorectal cancer. Oncogene (2013) 32:1910–20. 10.1038/onc.2012.214 PMC3630484 22710713

[33] De Cauwer A Mariotte A Sibilia J Bahram S Georgel P. DICER1: A Key Player in Rheumatoid Arthritis, at the Crossroads of Cellular Stress, Innate Immunity, and Chronic Inflammation in Aging. Front Immunol (2018) 9:1647. 10.3389/fimmu.2018.01647 30087677 PMC6066587

[34] Jiang C Xu J Zhu W Cai Y Wang S Guo Y. Abnormal Expression of DICER1 Leads to Dysregulation of Inflammatory Effectors in Human Synoviocytes. Mediators Inflammation (2019) 2019:6768504. 10.1155/2019/6768504 PMC6558604 31275058

[35] Alsaleh G Nehmar R Bluml S Schleiss C Ostermann E Dillenseger JP. Reduced DICER1 Expression Bestows Rheumatoid Arthritis Synoviocytes Proinflammatory Properties and Resistance to Apoptotic Stimuli. Arthritis Rheumatol (2016) 68:1839–48. 10.1002/art.39641 26882526

[36] Ren W Zhang X Li W Feng Q Feng H Tong Y. Exosomal miRNA-107 induces myeloid-derived suppressor cell expansion in gastric cancer. Cancer Manag Res (2019) 11:4023–40. 10.2147/CMAR.S198886 PMC6511657 31190980

