# Supplementary material for: MicroRNA-497 Reduction and Increase of Its Family Member MicroRNA-424 Lead to Dysregulation of Multiple Inflammation Related Genes in Synovial Fibroblasts With Rheumatoid Arthritis
Source: Front Immunol. 2021 Mar 26;12:619392. doi: 10.3389/fimmu.2021.619392 (PMC8034293; doi:10.3389/fimmu.2021.619392)
Supplement: Supplementary file 1 [file DataSheet_1.pdf]

Supplementary materials

Supplementary materials include patient and primer information, cytokine arrangement of cytokine antibody array, Tarbase and Targetscan database verification information, and gene literature summary.

Supplementary table 1:Schematic diagram of miR-15/107 gene family sequence

name	Accession number	sequence
hsa-miR-15a-5p	MIMAT0000068	U**AGCAGC**ACAUAAUGGUUUGUG
hsa-miR-16-5p	MIMAT0000069	U**AGCAGC**ACGUAAAUAUUGGCG
hsa-miR-103a-3p	MIMAT0000101	**AGCAGC**AUUGUACAGGGCUAUGA
hsa-miR-195-5p	MIMAT0000461	U**AGCAGC**ACAGAAAUAUUGGC
hsa-miR-107	MIMAT0000104	**AGCAGC**AUUGUACAGGGCUAUCA
hsa-miR-424-5p	MIMAT0001341	C**AGCAGC**AAUUCAUGUUUUGAA
hsa-miR-497-5p	MIMAT0002820	C**AGCAGC**ACACUGUGGUUUGU
hsa-miR-503-5p	MIMAT0002874	U**AGCAGC**GGGAACAGUUCUGCAG
hsa-miR-646	MIMAT0003316	A**AGCAGC**UGCCUCUGAGGC
hsa-miR-6838-5p	MIMAT0027578	A**AGCAGC**AGUGGCAAGACUCCU

Supplementary table 2: Patient characteristics

Patient	Gender	Age (years)	Disease duration (years)	RF (IU/ml)	ESR (mm/h)	CRP (mg/l)
OA1	Female	68	16	9	8	3
OA2	Female	71	18	4	9	9
OA3	Male	76	23	3	16	4
OA4	Female	72	12	7	17	8
OA5	Female	58	13	6	7	2
OA6	Male	71	13	5	12	9
OA7	Female	63	12	6	21	6
OA8	Male	72	18	7	7	10
RA1	Male	64	12	254	53	13
RA2	Female	48	10	131	51	62
RA3	Female	53	7	378	31	15
RA4	Female	51	9	63	45	45
RA5	Female	55	6	157	57	27
RA6	Male	69	11	59	53	22
RA7	Female	74	12	257	59	17
RA8	Female	62	10	212	43	18

RF, rheumatoid factor; ESR, erythrocyte sedimentation rate; CRP, C-reactive protein; CCP, cyclic citrullinated peptide

Anti-CCP antibody are all positive for RA patitents.

Supplementary table 3: MiRNA mimic and inhibitor sequence information

name	product	Sequence (from 5′ to 3′)
**miRNA NC**	Mimic	Sense UUCUCCGAACGUGUCACGUTT<br>Antisense ACGUGACACGUUCGGAGAATT
**miR-424-5p**	Mimic	Sense CAGCAGCAAUUCAUGUUUUGAA<br>Antisense CAAAACAUGAAUUGCUGCUGUU
**miR-497-5p**	Mimic	Sense CAGCAGCACACUGUGGUUUGU<br>Antisense AAACCACAGUGUGCUGCUGUU
**miRNA NC**	Inhibitor	CAGUACUUUUGUGUAGUACAA
**miR-424-5p**	Inhibitor	UUCAAAACAUGAAUUGCUGCUG
**miR-497-5p**	Inhibitor	ACAAACCACAGUGUGCUGCUG

NC: negative control

Supplementary table 4: siRNA sequence information

name	product	Sequence (from 5′ to 3′)
**NC siRNA**	siRNA	Sense UUCUCCGAACGUGUCACGUTT<br>Antisense ACGUGACACGUUCGGAGAATT
***DICER1* siRNA 1**	siRNA	Sense GGACCAUUUACUGACAGAATT<br>Antisense UUCUGUCAGUAAAUGGUCCTT
***DICER1* siRNA 2**	siRNA	Sense GGCCAUUGGACACAUCAAUTT<br>Antisense AUUGAUGUGUCCAAUGGCCTT
***DICER1* siRNA 3**	siRNA	Sense CCUCCUGGUUAUGUAGUAATT<br>Antisense UUACUACAUAACCAGGAGGTT

Supplementary table 5: Primer information

Gene symbol	Ta *(℃)	Sequences (from 5′ to 3′)	
**hsa-miR-15a-5p**	60	Forward	UAGCAGCACAUAAUGGUUUGUG
**hsa-miR-16-5p**	60	Forward	UAGCAGCACGUAAAUAUUGGCG
**hsa-miR-103a-3p**	60	Forward	AGCAGCAUUGUACAGGGCUAUGA
**hsa-miR-195-5p**	60	Forward	UAGCAGCACAGAAAUAUUGGC
**hsa-miR-107**	60	Forward	AGCAGCAUUGUACAGGGCUAUCA
**hsa-miR-424-5p**	60	Forward	CAGCAGCAAUUCAUGUUUUGAA
**hsa-miR-497-5p**	60	Forward	CAGCAGCACACUGUGGUUUGU
**hsa-miR-503-5p**	60	Forward	UAGCAGCGGGAACAGUUCUGCAG
**hsa-miR-646**	60	Forward	AAGCAGCUGCCUCUGAGGC
**hsa-miR-6838-5p**	60	Forward	AAGCAGCAGUGGCAAGACUCCU
**RNU6 (U6 small nuclear RNA)**	60	Forward	CTCGCTTCGGCAGCACA
		Reverse	AACGCTTCACGAATTTGCGT
**IL1B**	59	Forward	AGAAGTACCTGAGCTCGCCA
		Reverse	CTGGAAGGAGCACTTCATCTGT
**IL6**	61	Forward	CAATCTGGATTCAATGAGGAGAC
		Reverse	CTCTGGCTTGTTCCTCACTACTC
**IL8**	61	Forward	TGGAGAAGTTTTTGAAGAGGGCT
		Reverse	ACAGACCCACACAATACATGAAG
**MMP3**	59	Forward	AGTCTTCCAATCCTACTGTTGCT
		Reverse	TCCCCGTCACCTCCAATCC
**MMP13**	59	Forward	AATATCTGAACTGGGTCTTCCAAAA
		Reverse	CAGACCTGGTTTCCTGAGAACAG
**TGFB**	59	Forward	CTAATGGTGGAAACCCACAACG
		Reverse	TATCGCCAGGAATTGTTGCTG
**TLR3**	54	Forward	GCTAGCAGTCATCCAACAGAATC
		Reverse	AGTCAACTTCAGGTGGCTGC
**TLR4**	54	Forward	AATCTAGAGCACTTGGACCTTTCC
		Reverse	GGGTTCAGGGACAGGTCTAAAGA
**BCL2**	60	Forward	AGGCTGGGATGCCTTTGTGG
		Reverse	TTTGTTTGGGGCAGGCATGT
**TRIM23**	60	Forward	AACAAACAGGATGTTGCTGGAG
		Reverse	GAGCCAGTCCAACCCTTCAT
**SUMO3**	60	Forward	GAGAGGCAGGGCTTGTCAAT
		Reverse	CGGGCCCTCTAGAAACTGTG

**CEP55**	60	Forward	ACTTTTGGAGAAAATTCGAGTCCT
		Reverse	GTTGGTCTCTCAGTCGCTGT
**E2F1**	60	Forward	GCCATCCAGGAAAAGGTGTGA
		Reverse	GTGATGTCATAGATGCGCCG
**CCNA2**	60	Forward	CCAGGAGAATATCAACCCGGA
		Reverse	GGTGCAACCCGTCTCGT
**KCNN4**	60	Forward	CCGAGAGGCAGGCTGTTAAT
		Reverse	CAGACGATCTTGCCCCACAT
**GAPDH**	60	Forward	CACCCACTCCTCCACCTTTG
		Reverse	CCACCACCCTGTTGCTGTAG

*：Annealing Temperature

Supplementary table 6:
Cytokine arrangement of RayBio® C-Series human cytokine antibody array C5.

	A	B	C	D	E	F	G	H	I	J	K
**1**	POS	POS	POS	POS	NEG	NEG	ENA-78	G-CSF	GM-CSF	GRO	GRO-α
**2**	I-309	IL-1α	IL-1β	IL-2	IL-3	IL-4	IL-5	IL-6	IL-7	IL-8	IL-10
**3**	IL12-p40	IL-13	IL-15	IFN-γ	MCP-1	MCP-2	MCP-3	M-CSF	MDC	MIG	MIP-1β
**4**	MIP-1-	RANTES	SCF	SDF-1	TARC	TGF-β1	TNF-α	TNF-β	EGF	IGF-1	Angiogenin
**5**	OSM	TPO	VEGF	PDGF-BB	Leptin	BDNF	BLC	CK β8-1	Eotaxin	Eotaxin-2	Eotaxin-3
**6**	FGF-4	FGF-6	FGF-7	FGF-9	FLT-3 Ligand	Fractalkine	GCP-2	GDNF	HGF	IGFBP-1	IGFBP-2
**7**	IGFBP-3	IGFBP-4	IL-16	IP-10	LIF	LIGHT	MCP-4	MIF	MIP-3α	NAP-2	NT-3
**8**	NT-4	OPN	OPG	PARC	PIGF	TGF- b 2	TGF- b 3	TIMP-1	TIMP-2	POS	POS

Supplementary table 7:
Dysregulated genes in RASF after gain of miR-424 function detected by using mRNA deep sequencing

Gene Symbol	Regulation after transfected with mimic	padj	Function in literature	Outcome after transfected with mimic
**ZNF367**	Down	6.56E-08	ZNF367 knockdown increased proliferation in vitro and vivo, also increased cellular invasion and migration. [1]	Promote the proliferation
**BTRC**	Down	3.80E-08	BTRC is negative factors controlling IκB degradation in the type 1 TNF receptor (TNF-RI) and IL-1R pathways uponTNF-α and IL-1β stimulation. [2]	inhibit the degradation of IkB
**CCNA2**	Down	0.00832	CCNE2 or CCNA2 silencing markedly decreased abl cell and CWR22Rv1 cell growth. [3]	inhibit proliferation
**CD83**	Down	0.00162	In mixed leukocyte reaction (MLR), sCD83 inhibits DC-mediated T cell activation and proliferation.[4]	Promote the proliferation
**CDC6**	Down	0.01602	Down-regulation of CDC6 in HO8910 cells decreased cell proliferation and colony formation.[5]	inhibit proliferation
**CEP55**	Down	0.00328	knockdown of CEP55 markedly inhibited cell viability and proliferation and CEP55-silenced cells were obviously arrested in the G0/G1 phase and presented significant cell apoptosis .[6]	inhibit proliferation and promote apoptosis
			CEP55 knockdown significantly induced cell cycle arrest at G1 phase and suppressed OS cell proliferation, migration and invasion. [7]	inhibit proliferation
**E2F1**	Down	0.01955	E2F1 silencing effectively downregulated expression of canonical E2F target genes and suppressed RANKL-induced osteoclast genesis. [8]	Suppress osteoclast genesis
**E2F2**	Down	3.65E-05	In embryonic fibroblasts (MEF) of E2F2 knockout mice, the expression of IL-1α, IL-1β and TNF-α was suppressed. [9]	Upregulate the expression of IL-1α, IL-1β, and TNF-α
			Silencing E2F2 suppresses the proliferation, migration, and invasion of RASFs in vitro.[10]	inhibit proliferation
**KCNN4 (KCa3.1)**	Down	0.00240	Different concentrations of the KCa3.1 inhibitor significantly reduced the proliferation of RASFs. [11]	inhibit proliferation
**STMN1**	Down	0.00111	Inhibition of STMN1 showed a decrease in cell proliferation.[9]	inhibit proliferation

Supplementary table 8:
Dysregulated genes in RASF after gain of miR-497 function detected by using mRNA deep sequencing

Gene Symbol	Regulation after transfected with mimic	padj*	Function in literature	Outcome after transfected with mimic
**BCL2**	Down	1.10E-06	SF Treg cells transcribing high levels of Bcl-2 and microRNA-21 demonstrate limited apoptosis in RA[12].	Promote apoptosis
**TRIM23**	Down	8.16E-11	Overexpression of TRIM23 can activate the NF-κB pathway[13]	Inhibit NF-κB pathway
**SUMO3**	Down	1.28E-07	The knockdown of SUMO-2/3 significantly increased the TNF-α and IL-1β, induced expression of MMP-3 and MMP-13, accompanied by increased NF-κB activity[14].	Promote NF-kB pathway

* : p-value adjusted

Supplementary table 9: Validated miR-424 target genes from Tarbase.

Gene name	miRNA name	Experiments throughput	Cell lines	Tissues
PDCD4	hsa-miR-424-5p	low: 0 high: 6	BETA Cells, 293S, HEK293, HELA	Pancreas, Cervix, Kidney
BCL2	hsa-miR-424-5p	low: 0 high: 3	EF3DAGO2, HEK293, HELA	Cervix, Kidney
CCNE1	hsa-miR-424-5p	low: 3 high: 5	THP1, HEPG2, HELA, 293S, HEK293	Peripheral Blood, Liver, Cervix, Kidney
CCNE2	hsa-miR-424-5p	low: 1 high: 0	THP1	Peripheral Blood
CCND1	hsa-miR-424-5p	low: 2 high: 11	THP1, HEPG2, MDAMB231, HEK293,293S, HS5, HS27A	Mammary Gland, Peripheral Blood, Liver, Kidney, Bone Marrow
CCND3	hsa-miR-424-5p	low: 1 high: 0	HEPG2	Liver

Supplementary table 10: Validated miR-497 target genes from Tarbase.

Gene name	miRNA name	Experiments throughput	Cell lines	Tissues
**PDCD4**	hsa-miR-497-5p	low: 0 high: 8	BETA Cells, 293S, HELA	Pancreas, Cervix, Kidney, Brain
**BCL2**	hsa-miR-497-5p	low: 0 high: 2	EF3DAGO2, HELA	Cervix
**CCNE1**	hsa-miR-497-5p	low: 5 high: 3	HELA, 293S, MDAMB231, SKHEP1, HEPG2, HEK293T	Mammary Gland, Liver, Cervix, Kidney
**CCND1**	hsa-miR-497-5p	low: 5 high: 7	HEK293A, SGC7901, SKHEP1, HEPG2,293S, HS5, HS27A, HMSC	Gastric, Liver, Kidney, Bone Marrow
**CCND3**	hsa-miR-497-5p	low: 3 high: 0	SKHEP1, HEPG2	Liver

Supplementary table 11: Validated DICER1 and miRNA target relationship from Tarbase

Gene name	miRNA name	Experiments throughput	Cell lines	Tissues
**DICER1**	hsa-miR-424-5p	low: 1 high: 2	THP1, MDAMB231,293S	Peripheral Blood, Mammary Gland, Kidney
**DICER1**	hsa-miR-497-5p	low: 0 high: 3	293S	Kidney

References:

1 Jain, M. *et al.* ZNF367 inhibits cancer progression and is targeted by miR-195. *PloS one* **9**, e101423, doi:10.1371/journal.pone.0101423 (2014).

2 Mu, N. *et al.* A novel NF-kappaB/YY1/microRNA-10a regulatory circuit in fibroblast-like synoviocytes regulates inflammation in rheumatoid arthritis. *Scientific reports* **6**, 20059, doi:10.1038/srep20059 (2016).

3 Zhang, C. *et al.* Definition of a FoxA1 Cistrome that is crucial for G1 to S-phase cell-cycle transit in castration-resistant prostate cancer. *Cancer research* **71**, 6738-6748, doi:10.1158/0008-5472.CAN-11-1882 (2011).

4 Lechmann, M. *et al.* The extracellular domain of CD83 inhibits dendritic cell-mediated T cell stimulation and binds to a ligand on dendritic cells. *J Exp Med* **194**, 1813-1821, doi:10.1084/jem.194.12.1813 (2001).

5 Deng, Y. *et al.* High expression of CDC6 is associated with accelerated cell proliferation and poor prognosis of epithelial ovarian cancer. *Pathol Res Pract* **212**, 239-246, doi:10.1016/j.prp.2015.09.014 (2016).

6 Liu, L., Mei, Q., Zhao, J., Dai, Y. & Fu, Q. Suppression of CEP55 reduces cell viability and induces apoptosis in human lung cancer. *Oncology reports* **36**, 1939-1945, doi:10.3892/or.2016.5059 (2016).

7 Xu, L. *et al.* CEP55 promotes the proliferation and invasion of tumour cells via the AKT signalling pathway in osteosarcoma. *Carcinogenesis* **39**, 623-631, doi:10.1093/carcin/bgy017 (2018).

8 Murata, K. *et al.* Hypoxia-Sensitive COMMD1 Integrates Signaling and Cellular Metabolism in Human Macrophages and Suppresses Osteoclastogenesis. *Immunity* **47**, 66-79 e65, doi:10.1016/j.immuni.2017.06.018 (2017).

9 Wang, S., Wang, L., Wu, C., Sun, S. & Pan, J. H. E2F2 directly regulates the STAT1 and PI3K/AKT/NF-kappaB pathways to exacerbate the inflammatory phenotype in rheumatoid arthritis synovial fibroblasts and mouse embryonic fibroblasts. *Arthritis research & therapy* **20**, 225, doi:10.1186/s13075-018-1713-x (2018).

10 Zhang, R., Wang, L., Pan, J.-h. & Han, J. A critical role of E2F transcription factor 2 in proinflammatory cytokines-dependent proliferation and invasiveness of fibroblast-like synoviocytes in rheumatoid Arthritis. *Scientific reports* **8**, doi:10.1038/s41598-018-20782-7 (2018).

11 Friebel, K., Schonherr, R., Kinne, R. W. & Kunisch, E. Functional role of the KCa3.1 potassium channel in synovial fibroblasts from rheumatoid arthritis patients. *J Cell Physiol* **230**, 1677-1688, doi:10.1002/jcp.24924 (2015).

12 van der Geest, K. S. *et al.* SF Treg cells transcribing high levels of Bcl-2 and microRNA-21 demonstrate limited apoptosis in RA. *Rheumatology (Oxford)* **54**, 950-958, doi:10.1093/rheumatology/keu407 (2015).

13 Poole, E. *et al.* Identification of TRIM23 as a cofactor involved in the regulation of

NF-kappaB by human cytomegalovirus. *Journal of virology* **83**, 3581-3590, doi:10.1128/JVI.02072-08 (2009).

14 Frank, S. *et al.* Regulation of matrixmetalloproteinase-3 and matrixmetalloproteinase-13 by SUMO-2/3 through the transcription factor NF-kappaB. *Annals of the rheumatic diseases* **72**, 1874-1881, doi:10.1136/annrheumdis-2012-202080 (2013).
